# Anatomical site prevalence and genotypes of *Chlamydia trachomatis* infections among men who have sex with men: a multi-site study in China

**DOI:** 10.1186/s12879-019-4664-1

**Published:** 2019-12-10

**Authors:** Ying Zhou, Yu-Mao Cai, Shi-Liang Li, Ning-Xiao Cao, Xiao-Feng Zhu, Feng Wang, Yan Han, Yue-Ping Yin, Xiang-Sheng Chen

**Affiliations:** 1 Institute of Dermatology, Chinese Academy of Medical Science & Peking Union Medical College, Nanjing, China; 2 National Center for STD Control, Chinese Centers for Disease Control and Prevention, Nanjing, China; 3 Department of Dermatology, Affiliated Hospital of Guangdong Medical University, Zhanjiang, Guangdong China; 4 Shenzhen Center for Chronic Disease Control, Shenzhen, China; 5 Wuhan Institute of Dermatology, Wuhan, China

**Keywords:** *Chlamydia trachomatis*, Prevalence, Genotype, MSM, China

## Abstract

**Background:**

*Chlamydia trachomatis* (CT) infection is one of the most pervasive sexually transmitted infections and has high prevalence in urogenital and extra-urogenital sites among men who have sex with men (MSM). This study investigated anatomical site-specific prevalence and genotypes of CT among MSM recruited from three geographic areas in China.

**Methods:**

We collected urine specimens and anorectal, pharyngeal swab specimens from 379 MSM. CT infection was identified using polymerase chain reaction and CT genotyping was determined by sequences of the ompA gene.

**Results:**

The results indicated that the overall prevalence of CT infection was 18.2% (95% confidence intervals [CIs], 13.9–22.5%) and significantly different between the cities (*p* = 0.048). The infection was most common at the anorectal site (15.6, 95%CIs 11.6–19.5%) followed by urethral (3.2, 95%CIs 1.4–5.0%) and oropharyngeal sites (1.6, 95%CIs 0.3–2.9%). Genotypes D and G were the most common CT strains in this population but genotype D was significantly predominated in Nanjing while genotype G was in Wuhan. No genotype related to lymphogranuloma venereum was found. CT infection was significantly related to the infection of *Neisseria gonorrhoeae* (adjusted odds ratio [aOR] 14.27, 95%CIs 6.02–33.83, *p* < 0.001) and age. Men older than 40 years old were less likely to have a CT infection as compared to men under 30 years old (aOR 0.37, 95% CIs 0.15–0.93, *p* = 0.03).

**Conclusion:**

The high CT infection prevalence, particularly in the anorectal site, among MSM suggests the necessity to development an integrated CT screening and treatment program specifically focusing on this high-risk population. Surveillance of CT infections should be improved by including both infection and genotype based surveys into the current surveillance programs in China.

## Background

Globally, chlamydia, caused by *Chlamydia trachomatis* (CT), has become the most pervasive sexually transmitted infection (STI), representing a global prevalence of chlamydia of 4.2% in men aged 15–49 years [1]. Previous studies have confirmed that most MSM are asymptomatic. Untreated CT infections in men can lead to serious complications, including non-gonococcal urethritis, epididymitis and infertility [2, 3]. In addition, genital CT infections can significantly increase the risk of HIV transmission [4]. Although CT infections in China are not routinely reported as a notifiable infectious disease into the national surveillance program, data from the sentinel surveillance in 105 city-sized sites have indicated an increase of these infections from 32.5 per 100,000 in 2008 to 37.2 per 100,000 in 2015 [4]. In addition to a high prevalence of human immunodeficiency virus (HIV) infection [5], men who have sex with men (MSM) in China are likewise disproportionately affected by other STIs, including chlamydial infections. Acquisition of an STI among MSM is usually subject to the sexual behaviors (homosexual or bisexual behaviors) in this population, resulting in rectal and urethral infections mostly through insertive and receptive anal intercourses as well as possible transmission through oral-anal contact. Extended knowledge on prevalence and genotypes of CT infections among this population is of great importance. In addition to their value for providing background information for public health resource allocation and policy development, such studies would have a direct clinical relevance for improving control of CT infections by screening and treatment programs. We have used genotyping to study the genotype distribution of CT infection association with sex network, the transmission and LGV epidemic surveillance. An increasing number of studies on the prevalence and genotypes of CT infections have been conducted among this population in China [6–8], but previous studies conducted comprised mostly investigations of the infections on limited anatomical sites [6, 7] or among a sample of MSM who were recruited from a single geographic city [8]. Our study provided more comprehensive and accurate epidemiological information by including more geographical regions to improve geographic representativeness in China and more anatomical sites (rectum, urethra, and pharynx) to provide overall information of the infections in the study population. The current study was aimed to investigate CT infections among MSM by addressing (1) prevalence and genotypes by anatomical site (rectum, urethra, and pharynx) and geographic area; (2) genotype distribution of concurrent infections in multiple anatomical sites; and (3) factors associated with these infections.

## Methods

### Study area and population

This study was conducted in three cities located in three geographical regions, including Shenzhen in South China, Nanjing in East China and Wuhan in Central China. These cities are among the areas where the reported incidence of STIs is more than 50/100,000 [4] and a relatively high estimated size of MSM population in China [9]. The Shenzhen MSM population sample is from the Shenzhen Center for Chronic Disease Control. The outpatient service provides for the MSM population free syphilis and HIV screening and related consulting services. The collection of urine, pharyngeal swabs and anorectal swabs of the MSM population in Shenzhen was carried out in this institution. The Wuhan MSM population is from the Wuhan Institute of Dermatology. The department of prevention and treatment of STI is responsible for the general population and special high-risk groups for the screening, diagnosis, and treatment. It provides free HIV testing and treatment services for MSM people infected with HIV. The collection of urine, pharyngeal swabs and anorectal swabs of the MSM population in Wuhan was carried out in this population. The Nanjing MSM group: collection of urine, pharyngeal swabs and anorectal swabs of Nanjing MSM group was carried out on site at a gay bar. Four weekends were spent at the epidemiological survey site to collect relevant demographic and clinical information of the MSM population and collect urine, pharyngeal and anorectal swabs. We conducted enrollment of the participants from October 2017 to June 2018 in Shenzhen, from June to July 2018 in Nanjing and from April to August 2018 in Wuhan. All the samples were collected by trained medical professionals at the three centers. The inclusion criteria in this study included: male in gender, Chinese nationality, age more than 16 years, resident in the study area for more than 3 months, self-reported history of anal sex with another man in the past six months, willingness to participate in the study, and ability to provide a written informed consent, asymptomatic or symptomatic.

### Questionnaire and specimen collection

The eligible MSM were interviewed after they provided their written consent and completed a specifically designed, detailed socio-demographic and behavioral information form (Additional file 1). After completing the interview, the patients were then instructed by a trained nurse to collect 3–5 ml first-void urine specimen, while pharyngeal and rectal swabs were collected by the nurse at the clinic according to the standard operating procedure in the study protocol. The Cobas® urine specimen collection kit (Roche P/N 05170486190) was used to collect the urine samples and the Cobas® swab specimen collection kit (Roche P/N 05170516190) was used for the pharyngeal and rectal swab samples. These were collected according to the manufacturers’ instructions [10]. All samples were temporarily stored at 4 °C and then frozen at − 80 °C for a maximum of three months in a local laboratory. These were then sent to the central laboratory for testing.

### Laboratory assays

#### Identification of CT and *Neisseria gonorrhoeae* (NG)

The automated magnetism nucleic acid isolation method using the MagNA Pure 96 System (Roche Switzerland) was used to extract and purify the DNA from the urine and swab samples. This was done according to the manufacturer’s instructions. The extracted DNA was then analyzed for CT and NG based on the polymerase chain reaction (PCR) of the Cobas 4800® System using Cobas 4800® CT/NG Amplification/Detection Kit (Roche, Switzerland) [10]. The results judgement was automatic according to the preset computer program. The positive amplicons for CT or NG represented a CT or NG infection. DNA was extracted from the CT positive samples with the use of the DDH_2_O, Proteinase K and Buffer gA1 (TSINGKE, China), vortex oscillation 10 s and incubated for 15 min at 70 °C. DNA was obtained after purification and underwent nest PCR. Nest PCR uses two pairs (instead of one pair) of PCR primers to amplify the complete fragment. For the first run, the sample DNA is used as the template, while for the second run, the template is changed to the PCR product of the first run. The primer sequence, the PCR reaction system and the amplification program were provided by the MLST database website (http://mlstdb.bmc.uu.se) of the Uppsala University. The primers of ompA gene are shown in Table 1. The Qingke biotechnology company (TSINGKE, China) synthesized all primers. For the first run, the CT positive sample DNA was used as the template. The PCR reaction system was 30 μl, including I-5™ 2 x High Fidelity Master Mix (MCLAB, USA) 15 μl [11]; Outer primer F and R 1 μl, respectively; Template 1 μl, filled with water to 30 μl. Amplification procedure: pre-degeneration at 98 °C for 2 min, degeneration at 98 °C for 10 s, annealing at 55 °C for 10 s, stretching at 72 °C for 25 s for 30 cycles, 75 repair extension for 5 min, preservation at 4 °C. The PCR products were diluted 200 times. These products were the templates of the second run. The PCR reaction system and procedure of the second run were the same as those of the first run. The second PCR products with gel extraction were sequenced.

**Table 1:** Primer pairs used for PCR amplification and sequencing of ompA

*ompA*	118F	Outer primer	5′-ATTGCTACAGGACATCTTGTC-3′
	1163R	Outer primer	5′-CGGAATTGTGCATTTACGTGAG-3´
	ctr200F	Sequencing	5′-TTAGGIGCTTCTTTCCAATAYGCTCAATC-3´
	ctr254R	Sequencing	5′-GCCAYTCATGGTARTCAATAGAGGCATC-3´
	MOMP87	Inner primer	5′-TGAACCAAGCCTTATGATCGACGGA-3´
	RVS1059	Inner primer	5′-GCAATACCGCAAGATTTTCTAGATTTCATC-3´

#### Identification of ompA genotypes

The ompA sequence was compared using BLAST on the Pubmed website, and the ompA genotype of a CT strain was identified.

### Statistical analyses

To establish a database, one investigator entered the questionnaire data and laboratory results into Microsoft Office Excel forms. This was then checked by another investigator. The IBM SPSS Statistics for Windows Version 20.0 (IBM Corp., Armonk, NY) was used to statistically analyze the dataset. The independent variables were the study area, age group, education, marital status, local residency, sexual orientation, primary sexual role, number of sexual partners during past 6 months and gonococcal infection were included in the model. Outcome variables include overall and subgroup-stratified prevalence rates of CT infection and their 95% confidence intervals (CIs). For continuous variables, we calculated the median and the 25–75% interquartile range (IQR). For categorical variables, we calculated frequencies and proportions and identified the differences between groups using chi-square (χ^2^) or Fisher’s exact test. Overall and subgroup-stratified prevalence rates of CT infection and their 95% confidence intervals (CIs) were estimated. To calculate the odds ratio (OR) and its 95% CIs and *p* value, the univariate binominal regression analyses was used to determine the association between variables and the outcome of interest. Variables showed significant association between variables and the outcome (*p* ≤ 0.1) in univariate analyses were included in the multivariate regression model to explore the association of variables with the outcome. Using the model, adjusted odds ratio (aOR) and its 95% CIs were estimated. Values (*p* ≤ 0.05) were thought statistically significant.

## Results

### Participant characteristics

Of the 379 participants included for analysis, 102 (26.9%) were from Nanjing, 182 (48.0%) from Shenzhen and 95 (25.1%) from Wuhan. Median age of the included men was 30 years (IQR, 24–38) and most of them were single (75.9%) and resident locally for more than 2 years (77.1%) and the half (47.4%) followed high school or higher education. The study subjects reported different sexual behaviors (Table 2). Of note, the reported sexual behaviors, including sexual orientations, primary roles in sex and number of sexual partners, were significantly different among MSM recruited from the three study cities (*P* < 0.01).

**Table 2:** Baseline characteristics of participants recruited from the study cities

Variable	Nanjing *n* = 101 (%)^a^	Shenzhen *n* = 182 (%)^a^	Wuhan *n* = 95 (%)^a^	*P* value (χ^2^ test)
Age group (years)				
Younger than 30	74 (73.2)	72 (39.6)	36 (37.9)	< 0.01
30–40	13 (12.9)	74 (40.6)	34 (35.8)	
Elder than 40	14 (13.9)	36 (19.8)	25 (26.3)	
Education				
Secondary school or below	48 (48.0)	109 (59.9)	44 (46.3)	0.14
High School	21 (21.0)	34 (18.7)	20 (21.1)	
College or upper	31 (31.0)	39 (21.4)	31 (32.6)	
Marital status				
Single	83 (83.0)	132 (72.5)	66 (69.5)	0.08
Married	14 (14.0)	36 (19.8)	17 (17.9)	
Divorce	3 (3.0)	14 (7.7)	12 (12.6)	
Local residency				
2 years or shorter	35 (35.4)	36 (19.8)	13 (14.4)	< 0.01
Longer than 2 years	64 (64.6)	146 (80.2)	77 (85.6)	
Sexual orientation				
Homosexual	48 (47.5)	124 (68.1)	57 (60.6)	< 0.01
Bisexual	53 (52.5)	58 (31.9)	37 (39.4)	
Primary role in sex				
Receptive	0 (0.0)	47 (26.6)	33 (35.9)	< 0.01
Insertive	48 (47.5)	73 (41.2)	28 (30.4)	
Both receptive & insertive	53 (52.5)	57 (32.2)	31 (33.7)	
No. partners during past 6 months				
Less than 3	85 (84.2)	114 (62.6)	82 (86.3)	< 0.01
3 to 5	11 (10.9)	50 (27.5)	9 (9.5)	
More than 5	5 (5.0)	18 (9.9)	4 (4.2)	

^a^The sum of numbers in some subgroups was less than the total number of participants because some participants did not respond to the questions

### CT prevalence and genotype distribution

**Fig. 1:**
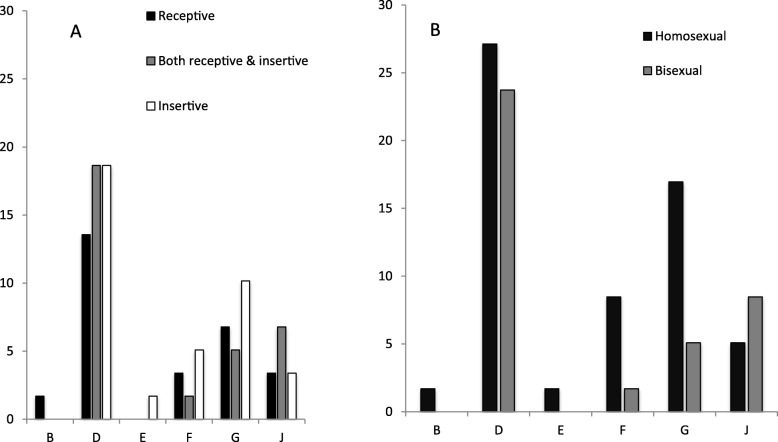
Distribution of genotypes of anorectal Chlamydia trachomatis strains among men who have sex with men who were diagnosed with chlamydial infection by role in sex (**a**) and sexual orientation (**b**)

The overall CT infection prevalence was 18.2% (95% CIs 13.9–22.5%) and CT/NG co-infection was 5.3% (95% CIs 3.0–7.6%). Anorectal CT infection was related to NG infection at the anorectal site (15.6% vs. 5.0%, *p* < 0.001), but this association was not found in urethral or pharyngeal site. Among MSM infected with NG, 68.4% (13/19), 0% (0/8) and 33.3% (1/3) had concurrent CT infection in anorectal, pharyngeal and urethral sites, respectively (p < 0.001). The overall prevalence rate of CT infection was statistically different across the cities. Shenzhen had a higher prevalence rate (22.5%) than Wuhan (10.5%) or Nanjing (17.6%), *p* = 0.048. The prevalence rate of anorectal CT infection (15.6, 95% CIs 11.6–19.5%) was significantly higher than urethral infection (3.2, 95% CIs 1.4–5.0%, *p* = 0.011) or pharyngeal infection (1.6, 95% CIs 0.3–2.9%, *p* = 0.226) (Table 3). Among the patients who were CT positive in any of the three anatomical sites, we successfully genotyped 77 specimens, including 59 anorectal, 6 pharyngeal and 12 urethral specimens. The most pervasive genotypes of CT were D (50.8%), G (22.0%), and F (10.2%) or J (13.6%) in anorectal infections. G and D were also predominant genotypes in either urethral or pharyngeal infections. However, there was a significant difference in genotype distribution across the cities (*p* < 0.001), indicating the most predominant genotype of G in Wuhan (60.0%) and D in Shenzhen (57.1%) or Nanjing (64.3%). The prevalence rate and genotype distribution stratified by sexual orientation and role in sex is shown in Fig. 1. Although different genotype distribution was found among MSM with different sexual orientations or roles in sex, the differences did not achieve statistical significance.

**Table 3:** Prevalence and odds of CT and NG infections by study area and anatomical site

City	Sample size	Chlamydia infection n; %(95% CIs*)								Gonococcal infection n; %(95% CIs*)							
		Total ^a^		Anorectal site		Pharyngeal site		Urethral site		Total		Anorectal site		Pharyngeal site		Urethral site	
Nanjing	102	18	17.6(11.0–26.7)	14	13.7(8.0–22.3)	4	3.9(1.3–10.3)	2	2(0.4–7.7)	8	7.8(3.7–15.3)	6	5.9(2.4–12.9)	2	2.0(0.4–7.7)	0	0
Shenzhen	182	41	22.5(16.8–29.4)	35	19.2(13.9–25.8)	2	1.1(0.2–4.3)	7	3.8(1.7–8.0)	14	7.7(4.4–12.8)	8	4.4(2.1–8.8)	4	2.2(0.7–5.9)	3	1.6(0.4–5.1)
Wuhan	95	10	10.5(5.4–18.9)	10	10.5(5.4–18.9)	0	0	3	3.2(0.8–9.7)	7	7.4(3.3–15.1)	5	5.3(2.0–12.5)	2	2.1(0.4–8.1)	0	0
Total	379	69	18.2(13.9–22.5)	59	15.6(11.6–19.5) ^b^	6	1.6(0.3–2.9)	12	3.2(1.4–5.0)	29	7.7(5.3–11.0)	19	5.0(3.1–7.8) ^c^	8	2.1(1.0–4.3)	3	0.8(0.2–2.5)

* *CIs*: confidence intervals

^a^ Statistically significant difference (*P* = 0.048, chi-square test) in proportions of chlamydia infection in Nanjing, Shenzhen and Wuhan

^b^ Statistically significant difference (*P* = 0.011, chi-square test) in proportions of chlamydia infection between anorectal site and urethral site

^c^ Statistically significant difference (*P* < 0.001,chi-square test) between proportions of anorectal chlamydia infection and proportions of anorectal gonococcal infection

### Factors associated with CT infection among men who have sex with men

In the univariate analysis, two variables (NG infection and age) showed significant associations with CT infection risk (*P* ≤ 0.1) (Table 4). In multivariate analysis, CT infection was significantly associated with NG infection (aOR 14.27, 95% CIs 6.02–33.83, *p* < 0.001) and age. Men older than 40 years old were less likely to have a CT infection as compared to men under 30 years old (aOR 0.37, 95% CIs 0.15–0.93, *p* = 0.03) (Table 4).

**Table 4:** Univariate and Multivariate analysis of associated factors with CT infection among men who have sex with men

Variable	Sample size	positive number (rate) n (%)	Univariate analysis		Multivariate analysis	
			OR (95% CIs)^a^	*P* value	Adjusted OR (95% CIs)^a^	*P* value
Chlamydial infection	379	69 (18.2)				
Age						
Younger than 30 years	182	38 (20.9)	Reference	─	Reference	─
30–40 years	121	24 (19.8)	0.94 (0.53–1.66)	0.83	1.01 (0.55–1.88)	0.96
Elder than 40 years	75	7 (9.3)	0.39 (0.17–0.92)	0.03	0.37 (0.15–0.93)	0.03
Gonococcal infection						
No	350	49 (14.0)	Reference	─	Reference	─
Yes	29	20 (69.0)	13.65 (5.88–31.70)	< 0.001	14.27 (6.02–33.83)	< 0.001

^a^*OR*: odds ratio; *CIs*: confidence intervals

### Factors associated with predominant anorectal genotypes (D, G) among anorectal CT infection of men who have sex with men

In the univariate analysis, two variables (study area and education) showed significant associations with anorectal genotype D infection risk (*P* ≤ 0.1) (Table 5). In multivariate analysis, the anorectal genotype D was significantly less observed in Wuhan MSM than in Nanjing (aOR 0.07, 95% CIs 0.01–0.71, *p* = 0.03) (Table 5). In the univariate analysis, two variables (study area and gonococcal infection) showed significant associations with anorectal genotype G infection risk (*P* ≤ 0.1) (Table 5). In multivariate analysis, we found no factors were significantly associated with genotype G rectal infection (Table 5).

**Table 5:** Univariate and Multivariate analysis of associated factors with predominant anorectal genotypes among anorectal CT infection of men who have sex with men

Variable	Sample size	positive number (rate) n (%)	Univariate analysis		Multivariate analysis	
			OR (95% CIs)^a^	*P* value	Adjusted OR (95% CIs)^a^	*P* value
Infection with genotype D	59	30 (50.8)				
Study area						
Nanjing	14	9 (64.3)	Reference	─	Reference	─
Shenzhen	35	20 (57.1)	0.74 (0.21–2.67)	0.65	0.66 (0.17–2.62)	0.56
Wuhan	10	1 (10.0)	0.06 (0.01–0.64)	0.02	0.07 (0.01–0.71)	0.03
Education						
Secondary school or below	34	20 (58.8)	Reference	─	Reference	─
High School	12	3 (25.0)	0.23 (0.05–1.02)	0.05	0.30 (0.06–1.44)	0.13
College or upper	13	7 (53.8)	0.82 (0.23–3.0)	0.76	1.00 (0.23–4.35)	1
Infection with genotype G	59	13 (22.0)				
Study area						
Nanjing	14	3 (21.4)	Reference	─	Reference	─
Shenzhen	35	4 (11.4)	0.47 (0.09–2.46)	0.37	0.47 (0.09–2.48)	0.38
Wuhan	10	6 (60.0)	5.50 (0.91–33.18)	0.06	4.50 (0.70–29.13)	0.11
Gonococcal infection						
No	43	7 (16.3)	Reference	─	Reference	─
Yes	16	6 (37.5)	3.09 (0.84–11.28)	0.09	1.80 (0.40–7.91)	0.44

^a^*OR*: odds ratio; *CIs*: confidence intervals

## Discussion

As far as we know, this study describes the maximum sample size of MSM recruited from multiple cities in China with CT infections and genotypes at multiple anatomical sites. Our findings show a high overall CT infection prevalence among MSM, which coincide with many other epidemiological researches in Kunming, China [12] or Bangkok, Thailand [13] reporting the prevalence of CT infection 14.3–18.2%. However we describe higher results than reported in some Western countries [14, 15]. The high prevalence was consistent across the study areas, indicating that the high-risk behaviors related to CT transmission among MSM may be present in different areas. They are generally unaware of their infections and unavailable of testing and treatment, resulting in untreated infections and further transmission [16]. The prevalence of anorectal CT infection was significantly higher than urethral or pharyngeal CT infection in our study population, which is consistent with many of the previous studies conducted in different regions in the world [14, 17, 18]. Our prevalence of anorectal CT infection (15.6%) was almost double that of the weighted average prevalence of the United States (9.0%) [19]. Although the results in Fig. 1 are not statistically significant, it is interesting that genotype E existed with an insertive sexual role. Since genotype E is the most epidemic among heterosexuals, we can infer from the genotype that this participant is most likely bisexual.

In our analysis, the anorectal CT genotype distribution indicates similar patterns with the findings from studies in Guangzhou, China [8] and in Melbourne and Sydney, Australia [20]. These studies found that the most frequent anorectal genotypes were D and G. The results are different from those in some European countries where the most frequent genotypes among MSM were L (49.2%) in the Netherlands [21] and E (37.5%) in Spain [22]. The urethral CT genotype distribution indicates similar patterns with the findings from studies in Shenzhen, China [6] and in Melbourne and Sydney, Australia [20]. These studies found that the most frequent urethral genotypes were D and G. No other pharyngeal sample and successful genotyping study was found for the pharyngeal site.

In our study, CT infection related to NG infection and the age of the MSM. MSM who had an NG infection had a 14.27 times higher CT infection risk than those without an NG infection. The relationship between CT infection and NG infection is further confirmed by our results [23]. China [24] and the USA CDC [25] both recommend that patients treated for an NG infection also should be treated with a regimen that is effective against uncomplicated genital CT infection and that patients who are diagnosed with a CT infection should be tested for NG. We found that MSM younger than 30 years had a higher risk of CT infection compared to MSM older than 40 years. These findings are similar to a study in Kunming, China [12]. This suggests that young MSM may have more unsafe sex because of the lack of the knowledge and awareness to protect against STIs. Thus, it is necessary to put more emphasis on the education of health relating to STIs among MSM and special efforts should be put to deliver services to young MSM. Our study also indicated that the genotype D was associated with the studied area. A previous study has indicated a high degree of concordance in transmission events between molecular and sexual network data [26]. However, it is not clear whether the difference in genotype distribution between the study areas was attributable to the different sexual network in these cities. Obviously, the background characteristics of sexual behaviors were different between the cities (Table 2). In addition, genotyping data in the Shenzhen MSM shows that the percentage of G strain was significantly lower than that reported in 2008–2009 (11.4% vs. 39%, *p* = 0.02) in the same clinic. Conversely, the percentage of D strain (57.1%) was higher than that found in 2008–2009 (37.0%) [6]. The genotype F accounting for 13.6% was found in the current study but not in the study conducted in 2008–2009 [6]. It is not clear whether this difference is related to the evolution of the predominant strains in the community over time or the change in behavioral or clinical characteristics. About one fifth (17%) of the Shenzhen participants in this study indicated that they were HIV positive whereas none of participants in the 2008–2009 study knew their HIV status or HIV negative [6].

The findings from the current study, combined with the previous studies in China [6–8], highlight the need for rectal and pharyngeal CT testing for MSM, in addition to screening for urethral infection. However, periodic testing for CT among MSM has not yet been included in the national STI management guidelines in China [24]. The World Health Organization (WHO) has recommended periodic detecting of rectal CT and NG infections among MSM and transgender women since 2011 [27]. The USA CDC recommends that an annual screening for urethral and anorectal infection of CT infection should be provided to men who have had insertive or receptive anal intercourse [25]. A lack of any recommendations for CT screening among high-risk groups in China and other resource-limited countries may be related to gaps in laboratory capacities and uncertainty about the cost-effectiveness [28]. Based on these considerations, simple, rapid and cheap diagnostics, including point-of-care (POC) tests, are important. Fortunately, there are several commercially available near-POC tests with acceptable performance characteristics and more are in the development pipeline [29]. In addition, a city-wide pilot study to validate programmatic strategies of CT infections screening among different populations including MSM in Shenzhen (the Shenzhen Chlamydia Intervention Pilot, SCIP) was started in early 2017. The findings from the pilot survey will be helpful for developing recommendations in China. No genotypes related to lymphogranuloma venereum (LGV) proctocolitis (L1, L2, or L3) were found in our study population although the outbreaks of this infection have been reported among MSM in several Western countries [30, 31]. This finding indicates that LGV strains have not yet spread among MSM during the period and in the area of the study in the MSM population [6]. However, given the frequent population movement across the continents, we should continue to supervise the appearance and spread of such strains in this high-risk population.

This study has some shortcomings. Firstly, this study was conducted at public health facilities, and a gay bar, which may lead to potential selection bias and may have an impact on the representativeness of the results. Secondly, information on sexual behaviors was self-reported during interview, which may lead to a self-report or social desirability bias and potential missing information on sexual behaviors on infections. Thirdly, the sample’s number was relatively small, particularly the total CT positive samples which were successfully genotyped were not sufficient for a statistical inference, which may restrict the generalization of the genotyping results. Fourthly, it should be mentioned that the time period to recruit the study population was different across the study sites. Such a difference might influence the study results. Finally, serum specimens were not specifically collected for this study to determine HIV and syphilis infection. Despite these limitations, our results may be very important for public health policy, clinical practice and epidemiological study.

## Conclusion

CT infection, particularly anorectal infections, is statistically prevalent among MSM in China. A comprehensive CT screening and treatment package focusing on this high-risk population is required in addition to currently promoting an integration of syphilis screening and treatment, and HIV prevention and control program among MSM in the country. Surveillance of CT infections should be improved by including both infection- and genotype-based surveys among high-risk groups, particularly MSM, as information from such surveys may be valuable to monitor the dynamical trends of sexual behaviors and transmission spread in the populations.

## Supplementary information

**Additional file 1.** Health Questionnaire.

## Data Availability

The results section, figures and tables contain all data on which the conclusions of this paper rely. The raw data used for this study are available from the corresponding author upon request.

## Abbreviations

CT: *Chlamydia trachomatis*
HIV: Human immunodeficiency virus
MSM: Men who have sex with men
STI: Sex transmitted infections
