# Supplementary material for: Anatomical site prevalence and genotypes of Chlamydia trachomatis infections among men who have sex with men: a multi-site study in China
Source: BMC Infect Dis. 2019 Dec 10;19:1041. doi: 10.1186/s12879-019-4664-1 (PMC6902585; doi:10.1186/s12879-019-4664-1)
Supplement: Supplementary file 1 — Additional file 1. Health Questionnaire. [file 12879_2019_4664_MOESM1_ESM.docx]

**Additional file 1**

| **Health Questionnaire** | | | | | | | |
| --- | --- | --- | --- | --- | --- | --- | --- |
| **Survey Setting：County City Province** | | | | | | | |
| **Source of samples：**① gay bar/dancing hall/teahouse/club ② bath/sauna/pedicure/massage   - ③ park/public toilet/lawn ④ network recruitment ⑤ others (please specify) | | | | | | | |
| **Number：** |  | | **Survey date：** | | | | |
| **Hello, we are conducting a survey to find out people's knowledge and behavior about some health problems. Please be assured that this survey is anonymous and your answers will be kept confidential. We also hope that your answer is your personal truth. The survey will take about 10 minutes of your time. At the end of the survey, we can provide you with free tests for *Chlamydia trachomatis* and *Neisseria gonorrhoeae* and provide you with some consultation on health problems. The results of the free test will be given to you within 1 month according to the information you provided. We hope you can support and assist us in this work. Thank you very much!** | | | | | | | |
| **Nickname** |  | **Phone number** |  | | **Nationality:** □Han □Other | | |
| **Age** |  | **Living place** | Street District | | **Syphilis TRUST：**□ Negative □Positive | | |
| **Marital Status** |  | **Domicile place** | City Province | | **Positive titer** | | 1： |
| **Education** |  | **Occupation** |  | | **Syphilis TPPA:** □ Negative □Positive | | |
| **History of blood donation** | □Yes □No | **History of drug use** | □Yes □No | **Local stay time** | | Year Month | |
| **HIV test** | □Yes □No | **HIV screen** | □Yes □No | **HIV conformation** | | □Negative □Positive □Not sure | |
| **Sexual orientation** | | □Homosexual □Bisexual | **Sexual role** | | | □Receptive □Insertive □Receptive and insertive | |
| **Condom use** | □Never use □Sometimes use □Always use | **Number of partners** | □Less than 3 partners  □3 to 5 partners □More than 5 partners | | **History of STIs** | □No □Yes | |
